# Visualization of the existence of growth hormone secretagogue receptor in the rat nucleus accumbens

**DOI:** 10.1186/s13041-024-01109-2

**Published:** 2024-06-13

**Authors:** Seohyeon Lee, Wen Ting Cai, Hyung Shin Yoon, Jeong-Hoon Kim

**Affiliations:** 1 Department of Medical Sciences, Yonsei University College of Medicine, Seoul, 03722 South Korea; 2 Department of Physiology, Yonsei University College of Medicine, Seoul, 03722 South Korea

**Keywords:** Nucleus accumbens, Growth hormone secretagogue receptor, Ghrelin receptor, Immunohistochemistry

## Abstract

The potential role of the ghrelin receptor, also known as the growth hormone secretagogue receptor (GHSR), within the nucleus accumbens (NAcc) in regulating drug addiction and feeding has been documented; however, the pattern of its expression in this site remains elusive. In this study, we characterized the expression patterns of GHSR1a and 1b, two subtypes of GHSRs, within the NAcc of the rat brain by immunohistochemistry. We visually detected GHSR signals, for the first time, at the protein level in the NAcc in which they were mostly expressed in neurons including both medium spiny neurons (MSNs) and non-MSNs. Furthermore, GHSR1a was found expressed as localized near the cellular membrane or some in the cytoplasm, whereas GHSR1b expressed solely throughout the large cytoplasmic area. The existence and subcellular expression pattern of GHSRs in the NAcc identified in this study will contribute to improving our understanding about the role of GHSR-mediated neurosignaling in feeding and drug addiction.

**Supplementary Information:**

The online version contains supplementary material available at 10.1186/s13041-024-01109-2.

The nucleus accumbens (NAcc) is a key region of the brain that mediates reward, addiction, and appetite [1]. Ghrelin is an orexigenic neuropeptide derived from the stomach [2]. In addition to its well-known role in food-related behaviors, it also has a regulatory role in drug addiction. Our previous study reported that ghrelin microinjections into the NAcc potentiated locomotor response to psychostimulants [3, 4], while these effects were diminished with antagonists of the ghrelin receptor, i.e., the growth hormone secretagogue receptor (GHSR), indicating that ghrelin acts primarily through GHSRs in the NAcc. Following a previous study demonstrating high levels of GHSR mRNA expression in the hippocampus or hypothalamus [5], it has been reported that GHSR mRNA is also expressed in the NAcc [6]. However, despite the evidence of mRNA existence, the exact expression pattern of the receptors at the protein level has not been shown yet in the NAcc and their specific localization in this site remains elusive [7], which hampers our understanding of GHSR-mediated neuronal mechanisms in this site. Thus, we aimed to visualize their expression at the protein level and determine their expression patterns at subcellular levels in the NAcc.

As GHSR consists of two subtypes, 1a and 1b [8], immunostaining was performed using antibodies specific to each subtype (Table S1). Detailed methods are described in Supplements. Fluorescent images obtained from the NAcc tissues with confocal microscopy clearly show that there exist two subtypes of GHSR expressed at the protein level in this site, with relatively stronger signals in the shell than in the core (Fig. 1A). To determine whether GHSR expression is in neuronal cells, double staining was performed using antibodies against the neuronal marker NeuN and antibodies for each subtype of GHSR. It is revealed that almost all GHSR signals were colocalized with NeuN markers across the NAcc subregions, indicating that the majority of GHSR is expressed in neuronal cells (Fig. 1B-E). As a negative control, it was verified that non-specific reactions did not occur in the NAcc tissues stained with no primary antibody (data not shown). Further, well-documented expression of GHSRs in the hippocampus was verified for the validity of our experimental conditions as a positive control (Fig. S1).

**Fig. 1:**
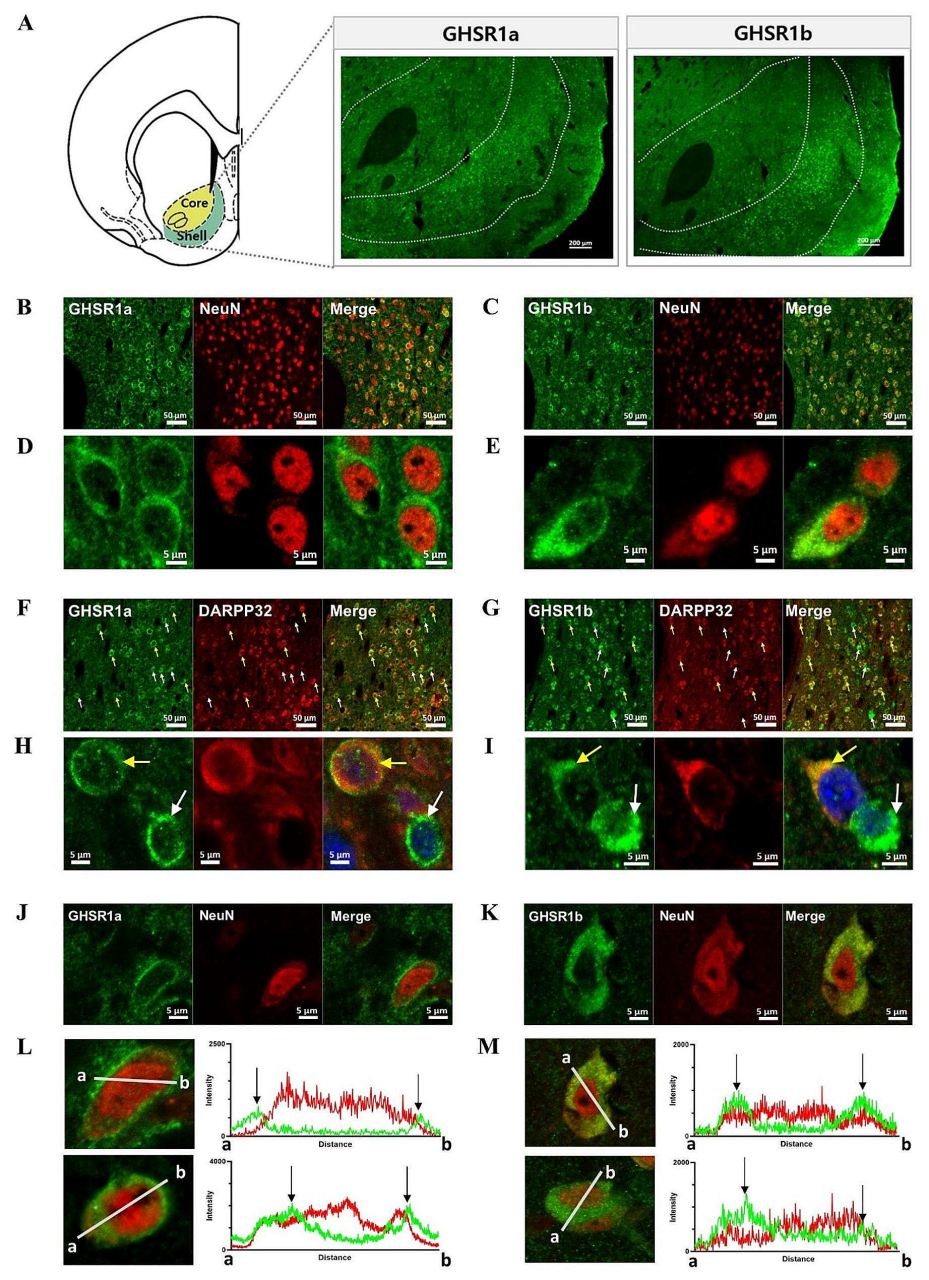
The expression profiles within the NAcc for two different subtypes of GHSRs. **A** Fluorescence signals of GHSR1a and GHSR1b were shown in the NAcc confirming the existence of GHSR at the protein level in this site (scale bar: 500 μm). The images were taken with 8 × 6 tiles at 20X magnification by confocal microscopy. Imaginary lines between the core and the shell were adopted from the Rat Brain Atlas of Paxinos and Watson (5th edition). **B-E** The images were taken at 20X (B, C; scale bar: 50 μm) or 63X magnification with zoom X3 (D, E; scale bar: 5 μm) by confocal microscopy. Almost all GHSR1a and GHSR1b were found expressed in neuron in the NAcc. **F-I** The images were taken at 20X (F, G; scale bar: 50 μm) or 63X magnification with zoom X3 (H, I; scale bar: 5 μm) by confocal microscopy. The majority of GHSR1a and GHSR1b signals were merged with dopamine- and cAMP-regulated neuronal phosphoprotein 32 kDa (DARPP-32) signals, a medium spiny neuron marker, in the NAcc. Yellow arrows indicate GHSRs expressed in the MSNs and white arrows indicate expression in non-MSNs. **J-M** The expression profiles of GHSR subtypes within a cell were analyzed using the Profile program of Zen 3.0. GHSR1a was observed expressed either as a circular form surrounding the cytoplasm in distal area of NeuN signaling or as uniformly spread to the cytoplasm, whereas GHSR1b was solely observed expressed uniformly distributed throughout the large cytoplasm area. The black arrows indicate the fluorescence peak signal of the GHSR.

Given that more than 95% of NAcc neurons are medium spiny neurons (MSNs) [9], we further explored whether GHSRs are expressed in MSNs using the MSN marker, dopamine- and cAMP-regulated neuronal phosphoprotein 32 kDa (DARPP-32). It is revealed that GHSRs were highly expressed in MSNs, with additional minor expression observed in non-MSNs (Fig. 1F-I). We further analyzed the ratios of GHSRs co-labelled with DARPP-32 out of total number of cells labelled either GHSRs or DARPP-32. When compared to total number of cells labelled with GHSRs, about 80 to 85% of them were found to exist in MSNs (as evidenced by co-labelling with DARPP-32), while the rest of them were in non-MSNs (Fig. S2A). Among GHSRs, GHSR1b is found slightly more labelled than GHSR1a in non-MSNs (15.2% vs. 20.4%). Looking into the total number of MSN cells labelled with DARPP-32, about 75% of them were found to express GHSR1a, while the ratio was increased to about 90% for those to express GHSR1b (Fig. S2B). The detailed raw scores used for calculation are shown in Table S2 in Supplements. Overall, it was observed that GHSR1b is more highly expressed than GHSR1a within MSNs as well as in non-MSN cells.

As it is reported that the subcellular localization of GHSR is significant for the understanding of their function [8], subsequent analysis for the subcellular localization of each GHSR subtype was performed by the Profile program of Zen 3.0 (Fig. 1J-M). First, a single cell was selected from the image, and the level of fluorescence signals was quantified at a cross-section along a vertical line traversing the nucleus (Fig. 1L, M). Two subtypes of GHSRs were all found uniformly distributed throughout the cytoplasm (Fig. 1D-M), whereas some GHSR1a interestingly appeared as distinctive circular form surrounding the cytoplasm area (Fig. 1D, H, J, L). When compared with NeuN-positive signals, GHSR1a was observed in distal part of and relatively less overlapped with them, whereas GHSR1b was observed more overlapped (Fig. 1L, M). While NeuN is long time known as being expressed localizing in the nucleus and perinuclear area, there are some evidence that it is also found expressed in cytoplasm area [10, 11]. Further, as an epitope of Rbfox3, a neuron-specific splicing regulator, turned out to be identified as NeuN, and it can be shifted from the nucleus to the cytoplasm area depending on its splice variants [12], it is not surprising for NeuN to be detected in the cytoplasm area. As we showed GHSRs were found overlapped with NeuN (Fig. 1L, M), we further examined whether it is localized in the cytoplasm area. By double staining cells in the NAcc with NeuN and α-tubulin, which is a cytoplasmic marker, we found that they were also co-localized (Fig. S3), indicating that NeuN is found expressed in even cytoplasmic area beyond nucleus and perinuclear area in the NAcc. Put together, GHSRs are found in the cytoplasmic area in the NAcc cells, with GHSR1a more likely localized near cellular membrane in distal portion of the NeuN-positive area (Fig. 1L), while GHSR1b localized throughout the cytoplasm near the nucleus (Fig. 1M).

We visually demonstrated, for the first time to the best of our knowledge, the expression of GHSR at the protein level in the NAcc, locating predominantly, in neurons including MSNs. Previously, it was observed in vitro that GHSR1a is present in both the cell membrane and cytoplasm [8] and GHSR1b is expressed mostly in the cytoplasm [13]. Consistent with these findings, our present findings in the NAcc indicate that GHSR1a is more likely found localized closer to the cell membrane as well as in the cytoplasm, whereas GHSR1b is distributed throughout the large area of the cytoplasm.

Approximately 95% of the NAcc neurons are MSNs [9], and it is well documented that reward-related behavioral modulation is associated with specific dopamine receptor subtypes expressed in these cells [14]. The remaining 5% of the NAcc neurons consist of cholinergic, fast-spiking GABA, and low-threshold spiking neurons [15]. Despite being in the minority, cholinergic neurons, for example, were recently found to influence reward-seeking behavior by affecting dopamine release [16, 17]. Our observation that GHSRs are present not only in MSNs but also in non-MSNs raises the possibility of their ability to regulate reward-related behaviors through multiple neurotransmitter signalings, including those involving dopamine and acetylcholine.

Previous studies have focused on GHSR1a to elucidate its function, based on the fact that it binds directly to its ligand and sends signals to the cell. In contrast, GHSR1b, a truncated variant of GHSR1a, has been considered to lack any signaling function for ghrelin. Interestingly, however, recent findings suggested that GHSR1b modulates the function of GHSR1a by changing the cellular location of GHSR1a and by making various combinations of GHSR1a/GHSR1b dimerization and a complex with dopamine receptors [18, 19]. Considering our previous report showing that direct application of ghrelin into the NAcc enhances locomotor activity to psychostimulants in a GHSR dependent manner [3, 4], these findings suggest that the ghrelin’s regulatory mechanisms in reward-related behaviors via GHSRs in this site are more complex than previously thought. The present findings that the NAcc has differential sub-cellular expression patterns for GHSR subtypes will provide deeper insights into the mechanism and function of the NAcc GHSR in regulating reward-related behaviors.

### Electronic supplementary material

Below is the link to the electronic supplementary material.

Supplementary Material 1

## Data Availability

The data presented in this study are available from the corresponding authors upon reasonable request.

## Abbreviations

GHSR1a: Growth hormone secretagogue receptor 1a
GHSR1b: Growth hormone secretagogue receptor 1b
NAcc: Nucleus accumbens
MSN: Medium spiny neuron
DARPP-32: Dopamine- and cAMP-regulated neuronal phosphoprotein 32 kDa
