# Supplementary material for: Visualization of the existence of growth hormone secretagogue receptor in the rat nucleus accumbens
Source: Mol Brain. 2024 Jun 13;17:37. doi: 10.1186/s13041-024-01109-2 (PMC11177452; doi:10.1186/s13041-024-01109-2)
Supplement: Supplementary file 1 — Supplementary Material 1 [file 13041_2024_1109_MOESM1_ESM.pdf]

Supplement

Visualization of the existence of growth hormone secretagogue receptor in the rat nucleus accumbens

Seohyeon Lee[1], Wen Ting Cai[2], Hyung Shin Yoon[2*] and Jeong-Hoon Kim[1,2*]

[1] Department of Medical Sciences, Yonsei University College of Medicine, Seoul 03722, South Korea

[2] Department of Physiology, Yonsei University College of Medicine, Seoul 03722, South Korea

* Correspondence:

Hyung Shin Yoon: hsyoon@yuhs.ac

Jeong-Hoon Kim: jkim1@yuhs.ac

This PDF file includes:

Supplementary Methods

Supplementary Tables: Table S1, S2

Supplementary Figures: Fig. S1, S2, S3

Supplementary Methods

1. Animals

Male Sprague-Dawley rats (200-230 g) were obtained from Orient Bio Inc. (Seongnam-si, Korea). Upon arrival, animals were housed in pairs under a 12-hour light/dark cycle, with a week allowed for acclimatization to their new environment before any experimental procedures began. Food and water were available ad libitum throughout the study. All procedures involving animals were reviewed and approved by the Institutional Animal Care and Use Committee of Yonsei University College of Medicine.

2. Perfusion, brain extraction and immunohistochemistry

Following acclimatization, rats were anesthetized with intraperitoneal injection of ketamine (100 mg/kg) and xylazine (6 mg/kg), followed by perfusion with saline and 4% paraformaldehyde (PFA). Brains were extracted, post-fixed with 4% PFA at 4°C overnight, and then submerged in 30% sucrose solution until they sank (3-4 days). The brains were embedded in OCT compound mixed with 30% sucrose, flash-frozen in isopentane on dry ice, sectioned coronally at 50 μm thickness. Sections ranging from 0.7 to 2.2 mm from bregma, as indicated in the Paxinos and Watson Rat Brain Atlas (5th ed.), were prepared for immunohistochemistry. Sections underwent 1-hour blocking in 10 mM PBS (pH 7.4) with 5% normal goat serum (Jackson ImmunoResearch Inc., West Grove, PA, USA) and 0.3% Triton X-100, followed by incubation at 4°C with primary antibodies in 10 mM PBS containing 2% normal goat serum and 0.1% Triton X-100. After washing 3 times (10 min each) in 10 mM PBS with 0.1% Triton X-100, sections were incubated with secondary antibodies for 2 hours at room temperature,

washed again, and mounted using Vectashield mounting medium with DAPI (Vector Laboratories, Burlingame, CA, USA) under a coverslip. The antibodies used are detailed in Table S1. It was verified that nonspecific reactions did not occur upon reaction with other NAcc tissues without a primary antibody (data not shown). The well-documented expression of GHSRs in the hippocampus from previous reports confirmed the validity of our experimental conditions (Fig. S1).

3. Confocal microscopy analysis

All images from the immunostained brain slices, including the NAcc, were acquired with LSM 700 confocal laser scanning microscope (Carl Zeiss) and analyzed using Zen 3.4 Software (Carl Zeiss, Jena, Germany).

Table S1. List of antibodies used in the experiment and their sources.

Target	Primary Antibody	Source	Secondary Antibody	Source
**GHSR 1a**	Rabbit polyclonal anti-GHSR1a (1:250)	Phoenix Pharmaceuticals Inc. (H-001-62)	Alexa 488 (1:2000)	abcam (ab150077)
**GHSR 1b**	Rabbit polyclonal anti-GHSR1b (1:250)	Phoenix Pharmaceuticals Inc. (H-001-61)	Alexa 488 (1:2000)	abcam (ab150077)
**NeuN**	Mouse monoclonal anti-NeuN (1:2000)	Abcam (ab104224)	Alexa 568 (1:2000)	abcam (ab175473)
**DARPP-32**	Mouse monoclonal anti-DARPP32 (1:1000)	BD Science (611520)	Alexa568 (1:2000)	abcam (ab175473)

Table S2. Counting immuno-labelled cells in the NAcc.

GHSR1a

Images (NAcc)	GHSR1a	DARPP-32	Merge	Total GHSR1a (GHSR1a+Merge)	Total MSN (DARPP-32+Merge)	GHSR1a in MSN (%) (Merge/total GHSR1a)	GHSR1a in non-MSN (%) (GHSR1a/total GHSR1a)	MSN with GHSR1a (%) (Merge/total MSN)	MSN without GHSR1a (%) (DARPP-32/total MSN)
Column 1	Column 2	Column 3	Column 4	Column 5	Column 6	Column 7	Column 8	Column 9	Column 10
Image 1 (core)	14	39	62	76	101	81.6	18.4	61.39	38.6
Image 2 (core)	25	32	79	104	111	76.0	24.0	71.17	28.8
Image 3 (core)	6	28	52	58	80	89.7	10.3	65.00	35.0
Image 4 (shell)	12	15	84	96	99	87.5	12.5	84.85	15.2
Image 5 (shell)	11	10	85	96	95	88.5	11.5	89.47	10.5
Image 6 (shell)	15	25	87	102	112	85.3	14.7	77.68	22.3
**Average**	**13.8**	**24.8**	**74.8**	**88.7**	**99.7**	**84.8**	**15.2**	**74.9**	**25.1**

GHSR1b

Images (NAcc)	GHSR1b	DARPP-32	Merge	Total GHSR1b (GHSR1b+Merge)	Total MSN (DARPP-32+Merge)	GHSR1b in MSN (%) (Merge/total GHSR1b)	GHSR1b in non-MSN (%) (GHSR1b/total GHSR1b)	MSN with GHSR1b (%) (Merge/total MSN)	MSN without GHSR1b (%) (DARPP-32/total MSN)
Column 1	Column 2	Column 3	Column 4	Column 5	Column 6	Column 7	Column 8	Column 9	Column 10
Image 1 (core)	18	18	74	92	92	80.4	19.6	80.43	19.6
Image 2 (core)	18	7	91	109	98	83.5	16.5	92.86	7.1
Image 3 (core)	31	21	108	139	129	77.7	22.3	83.72	16.3
Image 4 (shell)	24	7	106	130	113	81.5	18.5	93.81	6.2
Image 5 (shell)	32	5	97	129	102	75.2	24.8	95.10	4.9
Image 6 (shell)	26	5	98	124	103	79.0	21.0	95.15	4.9
**Average**	**24.8**	**10.5**	**95.7**	**120.5**	**106.2**	**79.6**	**20.4**	**90.2**	**9.8**

Table S2. A total of six images double-stained with GHSR and DARPP-32 antibodies were randomly selected and analyzed for each GHSR subtype. Neuronal cells were counted for labelling with DARPP-32 alone, GHSR alone, and both. Once total cell numbers labelled with GHSRs and DARPP-32 were obtained, the percentages of MSN and non-MSN showing GHSR immuno-reactivity out of total GHSR-labelled cells were calculated as shown in the 7 and 8 columns in the Table. Likewise, the percentage of MSN showing GHSR immuno-reactivity out of total MSN cells was calculated as shown in the 9 and 10 columns in the Table.

Fig. S1

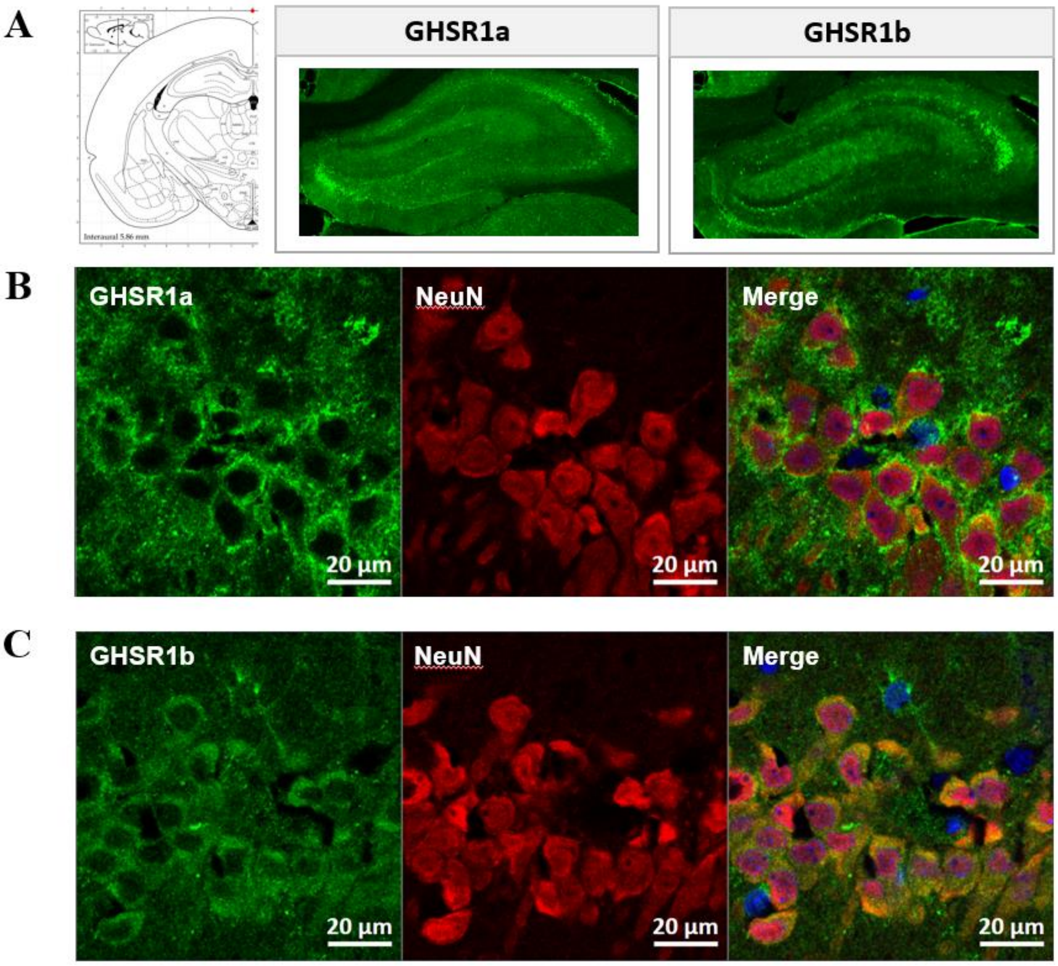

Fig. S1. The expression of GHSR1a and GHSR1b in the hippocampus of rats. **A** The experimental conditions used in the present study were validated by confirming the fluorescent signals of GHSRs in the hippocampus, where their presence is well known from previous studies. Representative images of the hippocampus sections were obtained from 8X10 tile scan with 20X magnification by confocal microscopy. **B, C** Representative images illustrate major expression profiles of GHSR1a and GHSR1b, respectively. GHSR1a was found in the proximal part of the plasma membrane as well as in the cytoplasm of neurons. GHSR1b, on the other hand, was predominantly distributed inside the cytoplasm of neurons (40X magnification by confocal microscopy; scale bar: 20 µm).

Fig. S2

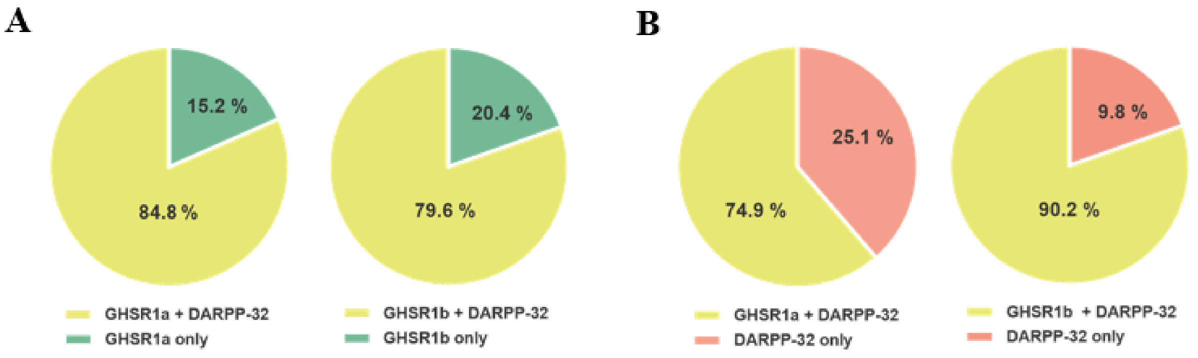

Fig. S2. Analysis for the ratios of GHSR expression between medium spiny neurons (MSNs) and non-MSNs and within MSNs in the NAcc. **A** Among the GHSR-labelled cells in the NAcc, about 80 to 85% of them were expressed in MSNs (co-labelled with DARPP-32 as shown in yellow part of the diagram), while about 15 to 20% of them were in non-MSNs (GHSR only with no DARPP-32 as shown in green part). **B** Within the MSNs in the NAcc, approximately 75 to 90% of cells were found to express GHSR (co-labelled with GHSR1a or 1b as shown in yellow part of the diagram), whereas GHSR expression was not detected in about 10 to 25% of cells (labelled with DARPP-32 only as shown in coral pink part of the diagram). Overall, it was observed that GHSR1b is more highly expressed than GHSR1a in non-MSN cells as well as within MSNs.

Fig. S3

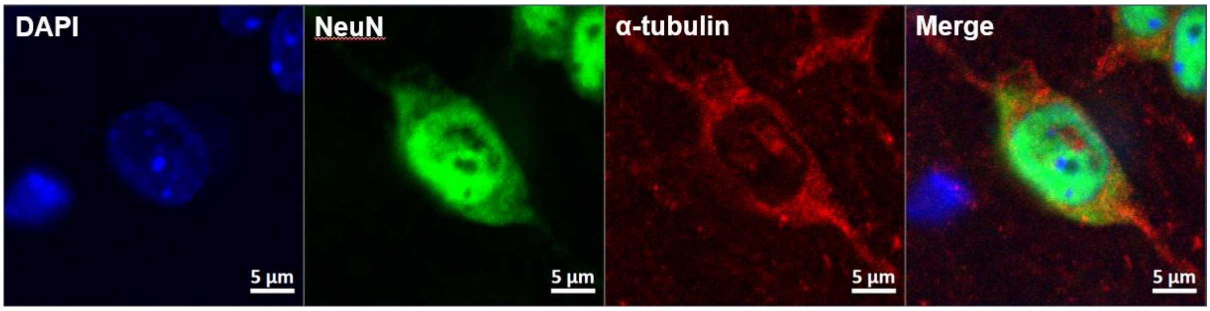

Fig. S3. Co-expression of NeuN and α-tubulin in the NAcc of rats. Fluorescence signals of NeuN and α-tubulin were shown in the NAcc confirming the expression of NeuN in the cytoplasm area. The images were taken at 63X magnification with zoom X3 (scale bar: 5 µm) by confocal microscopy. While α-tubulin was also found expressed in dendrites, NeuN was not co-expressed in such processes, and rather its expression was limited in the proximal part of these processes.
